# Cranberries for preventing urinary tract infections

**DOI:** 10.1590/1516-3180.20131315T1

**Published:** 2013-10-01

**Authors:** Ruth G. Jepson, Gabrielle Williams, Jonathan C. Craig

## Abstract

**BACKGROUND:**

Cranberries have been used widely for several decades for the prevention and treatment of urinary tract infections (UTIs). This is the third update of our review first published in 1998 and updated in 2004 and 2008.

**OBJECTIVES:**

To assess the effectiveness of cranberry products in preventing UTIs in susceptible populations.

**METHODS:**

*Search methods:* We searched MEDLINE, EMBASE, the Cochrane Central Register of Controlled Trials (CENTRAL in The Cochrane Library) and the Internet. We contacted companies involved with the promotion and distribution of cranberry preparations and checked reference lists of review articles and relevant studies.

*Date of search:* July 2012.

*Selection criteria:* All randomised controlled trials (RCTs) or quasi-RCTs of cranberry products for the prevention of UTIs.

*Data collection and analysis:* Two authors independently assessed and extracted data. Information was collected on methods, participants, interventions and outcomes (incidence of symptomatic UTIs, positive culture results, side effects, adherence to therapy). Risk ratios (RR) were calculated where appropriate, otherwise a narrative synthesis was undertaken. Quality was assessed using the Cochrane risk of bias assessment tool.

**MAIN RESULTS:**

This updated review includes a total of 24 studies (six cross-over studies, 11 parallel group studies with two arms; five with three arms, and two studies with a factorial design) with a total of 4473 participants. Ten studies were included in the 2008 update, and 14 studies have been added to this update. Thirteen studies (2380 participants) evaluated only cranberry juice/concentrate; nine studies (1032 participants) evaluated only cranberry tablets/capsules; one study compared cranberry juice and tablets; and one study compared cranberry capsules and tablets. The comparison/control arms were placebo, no treatment, water, methenamine hippurate, antibiotics, or lactobacillus. Eleven studies were not included in the meta-analyses because either the design was a cross-over study and data were not reported separately for the first phase, or there was a lack of relevant data. Data included in the meta-analyses showed that, compared with placebo, water or not treatment, cranberry products did not significantly reduce the occurrence of symptomatic UTI overall (RR 0.86, 95% CI 0.71 to 1.04) or for any the subgroups: women with recurrent UTIs (RR 0.74, 95% CI 0.42 to 1.31); older people (RR 0.75, 95% CI 0.39 to 1.44); pregnant women (RR 1.04, 95% CI 0.97 to 1.17); children with recurrent UTI (RR 0.48, 95% CI 0.19 to 1.22); cancer patients (RR 1.15 95% CI 0.75 to 1.77); or people with neuropathic bladder or spinal injury (RR 0.95, 95% CI: 0.75 to 1.20). Overall heterogeneity was moderate (I² = 55%). The effectiveness of cranberry was not significantly different to antibiotics for women (RR 1.31, 95% CI 0.85, 2.02) and children (RR 0.69 95% CI 0.32 to 1.51). There was no significant difference between gastrointestinal adverse effects from cranberry product compared to those of placebo/no treatment (RR 0.83, 95% CI 0.31 to 2.27). Many studies reported low compliance and high withdrawal/dropout problems which they attributed to palatability/acceptability of the products, primarily the cranberry juice. Most studies of other cranberry products (tablets and capsules) did not report how much of the 'active' ingredient the product contained, and therefore the products may not have had enough potency to be effective.

**AUTHORS' CONCLUSIONS:**

Prior to the current update it appeared there was some evidence that cranberry juice may decrease the number of symptomatic UTIs over a 12 month period, particularly for women with recurrent UTIs. The addition of 14 further studies suggests that cranberry juice is less effective than previously indicated. Although some of small studies demonstrated a small benefit for women with recurrent UTIs, there were no statistically significant differences when the results of a much larger study were included. Cranberry products were not significantly different to antibiotics for preventing UTIs in three small studies. Given the large number of dropouts/withdrawals from studies (mainly attributed to the acceptability of consuming cranberry products particularly juice, over long periods), and the evidence that the benefit for preventing UTI is small, cranberry juice cannot currently be recommended for the prevention of UTIs. Other preparations (such as powders) need to be quantified using standardised methods to ensure the potency, and contain enough of the 'active' ingredient, before being evaluated in clinical studies or recommended for use.

**This is the abstract of a Cochrane Review published in the Cochrane Database of Systematic Reviews (CDSR) 2012, issue 10, Art. No. CD001321. DOI:** 10.1002/14651858.CD001321.pub5 (http://cochrane.bvsalud.org/cochrane/main.php?lib=COC&searchExp=Cranberries%20and%20for%20and%20preventing%20and%20urinary%20and%20tract%20and%20infections&lang=pt). For full citation and authors details, see reference 1.

The abstract (English and Portuguese language) is available from: http://onlinelibrary.wiley.com/doi/10.1002/14651858.CD001321.pub5/abstract
